# Immediate and early percutaneous coronary intervention in very high‐risk and high‐risk non‐ST segment elevation myocardial infarction patients

**DOI:** 10.1002/clc.23781

**Published:** 2022-03-09

**Authors:** Lior Lupu, Louay Taha, Ariel Banai, Hezzy Shmueli, Ariel Borohovitz, Shlomi Matetzky, Mustafa Gabarin, Mony Shuvy, Roy Beigel, Katia Orvin, Sa'ar Minha, Yacov Shacham, Shmuel Banai, Michael Glikson, Elad Asher

**Affiliations:** ^1^ Department of Cardiology, Tel Aviv Sourasky Medical Center and Sackler Faculty of Medicine Tel Aviv University Tel Aviv Israel; ^2^ Department of Cardiology, Jesselson Integrated Heart Center, Shaare Zedek Medical Center and Faculty of Medicine Hebrew University of Jerusalem Israel; ^3^ Department of Cardiology, Soroka Medical Center, Beer‐Sheva, Israel and Faculty of Health Sciences Ben‐Gurion University of the Negev Beer‐Sheva Israel; ^4^ Department of Cardiology, Sheba Medical Center, Ramat Gan, Israel and Sackler Faculty of Medicine Tel Aviv University Tel Aviv Israel; ^5^ Dep­artment of Cardiology, Meir Medical Center, Kfar Saba, Israel and Sackler Faculty of Medicine Tel Aviv University Tel Aviv Israel; ^6^ Department of Cardiology, Rabin Medical Center, Petah Tikva, Israel and Sackler Faculty of Medicine Tel Aviv University Tel Aviv Israel; ^7^ Department of Cardiology, Shamir Medical Center, Be'er Ya'akov, Israel and Sackler Faculty of Medicine Tel Aviv University Tel Aviv Israel

**Keywords:** acute coronary syndrome (ACS), guidelines, non‐ST‐elevation myocardial infarction (NSTEMI), percutaneous coronary intervention (PCI)

## Abstract

**Background:**

The European Society of Cardiology (ESC) guidelines for the management of acute coronary syndromes in patients presenting without persistent ST‐segment elevation (non‐ST‐segment elevation myocardial infarction [NSTEMI]) has recommended immediate (<2 h) percutaneous coronary intervention (PCI) in very‐high risk patients and early (<24 h) PCI in high‐risk patients.

**Hypothesis:**

To examine the ESC NSTEMI guidelines adherence in a nationwide survey in Israel using the Acute Coronary Syndrome Israeli Survey (ACSIS). We hypothesized that adherence to the guidlines' recommnded PCI timing in NSTEMI pateints will be inadequate, partly due to the inconsistent evidence regarding its effect on clinical outcomes.

**Methods:**

All NSTEMI patients who underwent PCI during the ACSIS surveys in 2016 and 2018 were included in the analysis.

**Results:**

Out of 1793 NSTEMI patients, 1643 (92%) patients underwent PCI, and door to balloon time was documented in 1078 of them. One hundred and fifty‐six (14.5%) patients and 922 (85.5%) patients were defined as very high‐risk and high‐risk NSTEMI patients, respectively. Of the very high‐risk NSTEMI patients, only 10 (6.4%) underwent immediate coronary angiography, and 50 (32.1%) underwent early coronary angiography. Acute heart failure 139 (89.1%) was the main reason for including NSTEMI patients in the very high‐risk category. Of the high‐risk patients, early coronary angiography was performed in only 405 (43.9%) patients. Patients in whom coronary angiography was postponed were older and had more comorbidities.

**Conclusions:**

Despite guidelines recommendations for immediate and early PCI in very high‐risk and high‐risk NSTEMI patients, respectively, most patients do not undergo immediate or early PCI according to contemporary guidelines. Further studies are needed to better understand the reasons for guidelines' nonadherence in those high‐risk patients.

## 1 BACKGROUND

Acute coronary syndrome (ACS) is the acute form of ischemic heart disease, which is the leading cause of death globally.^1^ This definition of ACS ranges from ST‐segment elevation myocardial infarction (STEMI) to non‐STEMI (NSTEMI) and unstable angina (UA).^2^ Immediate primary percutaneous intervention (PCI) is the gold standard for the treatment of STEMI.^3^, ^4^ By contrast, routine use of coronary angiography, and its timing, in NSTEMI patients is still debatable.^5^ Several meta‐analyses support the role of a routine early (<24 h) PCI in reducing the risk of composite ischemic outcomes, particularly in high‐risk patients.^6^, ^7^, ^8^, ^9^ Moreover, randomized controlled trials have shown that very high‐risk and high‐risk NSTEMI patients may benefit from an early invasive strategy.^10^, ^11^, ^12^ Accordingly, the last two European Society of Cardiology (ESC) guidelines (2015, 2020) for the management of acute coronary syndromes in patients presenting without persistent ST‐segment elevation (NSTEMI) recommend an immediate and early routine PCI in very high‐risk and high‐risk NSTEMI patients, respectively.^13^, ^14^ We aim to examine adherence to these recommendations in a nationwide survey.

## 2 METHODS

Patients were derived from the ACS Israeli Survey (ACSIS). Details about this registry have been previously reported.^15^ In brief, the ACSIS is a nationwide survey conducted during March and April of 2016 and 2018 in all 25 cardiac units and cardiology wards operating in Israel. Local ethics committee approval was received from each hospital. Participants provided their written informed consent to participate in the study. The study population comprised all patients admitted with ACS.

Prespecified forms were used to collect demographic and clinical data. The discharge diagnoses were determined by the attending physician based on clinical, electrocardiogram, and biochemical tests. Patients' treatment was determined by the decision of the attending physician.

### 2.1 Study population

The study population consisted of patients included in the ACSIS surveys in 2016 and 2018 with the diagnosis of NSTEMI, which was defined according to contemporary guidelines.^13^ Patients were considered to have NSTEMI if they had acute chest discomfort with no persistent ST‐segment elevation and elevated troponin. The ECG findings ranged from normal ECG to changes that may include transient ST‐segment elevation, persistent or transient ST‐segment depression, T‐wave inversion, flat T waves, or pseudonormalization of T waves. Patients' risk stratification was performed according to the 2015 ESC guidelines.^13^ Very high‐risk patients were defined when they had one or more of the following: hemodynamic instability or cardiogenic shock; ongoing or recurrent pain refractory to medical treatment; life‐threatening arrhythmias or cardiac arrest; mechanical complications; or an acute heart failure. High‐risk patients were defined by one or more of the following: rise or fall in troponin compatible with myocardial infarction (MI); dynamic ST‐segment or T‐wave changes; or GRACE score higher than 140. The investigators who determined the risk category were blinded to clinical outcomes. Time to coronary angiography was divided into three categories: immediate coronary angiography was done <2 h from admission; early coronary angiography was done <24 h from admission; and late coronary angiography was done for >24 h from admission. We defined 30‐days major cardiovascular adverse events (MACE) as the occurrence in 30 days of either: mortality, UA pectoris, MI, stent thrombosis, urgent revascularization, and cerebrovascular event.

### 2.2 Statistical analysis

Patients' characteristics were presented as numbers (%) for categorical variables and as means (SD) or medians (IQR) for normal and nonnormal distributed continuous variables, respectively.

A *χ* ^2^ test for trends was used for the comparison of categorical variables. Analysis of variance with 1 degree of freedom was performed for comparison of normally distributed continuous variables and Kendall rank correlation for nonnormal distribution.

For MACE and 1‐year mortality outcomes, univariable and multivariable logistic regressions were performed with prespecified covariates. All covariates have less than 5% missing data except for family history of coronary artery disease, which was not included in the multivariable model.

An interaction term (PCI time × Risk class) was assessed, unadjusted for other covariates. Models were assessed among high‐risk and very high‐risk patients separately.

All tests were conducted at a two‐sided overall 5% significance level (*α* = .05).

All analyses were performed using R software (R Development Core Team, version 4.0.3).

## 3 RESULTS

A total of 1793 patients were diagnosed with NSTEMI during the study period. Coronary angiography was performed in 1643 (92%) patients. Time to intervention was documented in 1078 patients. Of them, 156 (14.5%) and 922 (85.5%) patients were defined as very high‐risk and high‐risk NSTEMI patients, respectively. Acute heart failure was the main reason (89.1%) for including patients in the very high‐risk NSTEMI category (Table S1).

### 3.1 Intergroup analyses

Patients in the very high‐risk group were older and with a higher proportion of women as compared with the high‐risk group. Furthermore, these patients had more comorbidities, including hypertension, hyperlipidemia, active smoking status, diabetes mellitus, family history of ischemic heart disease, history of chronic kidney disease, peripheral vascular disease, cerebrovascular disease, history of MI, PCI, coronary artery bypass grafting (CABG), congestive heart failure (CHF), and a higher proportion of GRACE score > 140 (Table 1).

**Table 1:** Baseline patients' characteristics

	Very high risk	High risk	*p* value
*n*	156	922	
*Baseline characteristics*			
Age, years (median [IQR])	68.50 (64.00, 77.00)	65.00 (56.00, 73.00)	<.001
Gender (male)	106 (67.9)	741 (80.4)	.001
Dyslipidemia	119 (76.8)	708 (76.8)	1
Hypertension	134 (86.5)	625 (67.8)	<.001
Current smokers	43 (27.6)	355 (38.5)	.011
Diabetes mellitus	95 (60.9)	394 (42.8)	<.001
Family history of CAD	26 (21.5)	254 (34.1)	.008
BMI (kg/m^2^), (median [IQR])	27.26 (24.67, 31.59)	27.46 (24.61, 30.25)	.771
Prior MI	84 (54.5)	379 (41.2)	.003
Prior CABG	25 (16.1)	97 (10.5)	.057
Prior PCI	72 (47.1)	331 (36.0)	.012
Chronic renal failure	45 (29.0)	87 (9.4)	<.001
Peripheral vascular disease	23 (14.8)	55 (6.0)	<.001
Cerebrovascular disease	30 (19.2)	74 (8.0)	<.001
CHF	37 (23.9)	66 (7.2)	<.001
GRACE score > 140	52 (36.4)	120 (13.5)	<.001

Abbreviations: BMI, body mass index; CABG, coronary artery bypass grafting; CAD, coronary artery disease; CHF, congestive heart failure; MI, myocardial infarction; PCI, percutaneous coronary intervention.

#### 3.1.1 Outcomes

No difference was found in the PCI rate during the index hospitalization between the groups. However, patients in the very high‐risk group had a higher number of diseased vessels and had a higher proportion of left main (15.5% vs. 3.3%, respectively; *p *< .001) and left anterior descending (LAD) (61.2% vs. 48.9%, respectively; *p* = .029) arteries PCI. Moreover, patients in the very high‐risk group had lower ejection fractions (EFs) (Table 2).

**Table 2:** Clinical outcomes in all cohort

	Very high risk	High risk	*p* value
*n* (%)	156	922	
*Reperfusion therapy*			
PCI	103 (66.0)	615 (66.7)	.941
Angiography	156 (100.0)	922 (100.0)	NA
*Number of diseased vessels*			<.001
None	3 (1.9)	46 (5.0)	
1 vessel	34 (21.8)	284 (31.0)	
2 vessels	41 (26.3)	298 (32.5)	
3 vessels	78 (50.0)	288 (31.4)	
PCI to LM	16 (15.5)	20 (3.3)	<.001
PCI to LAD	63 (61.2)	301 (48.9)	.029
PCI to LCX	33 (32.0)	215 (35.0)	.642
PCI to RCA	30 (29.1)	197 (32.0)	.636
PCI to SVG	4 (4.1)	17 (3.0)	.781
PCI to arterial graft	0 (0.0)	2 (0.4)	1
*EF classes*			<.001
Normal (EF > 50%)	59 (41.8)	513 (66.9)	
Mild (EF: 40%–50%)	29 (20.6)	178 (23.2)	
Moderate (EF: 30%–40%)	32 (22.7)	61 (8.0)	
Severe (EF < 30%)	21 (14.9)	15 (2.0)	
*30‐day clinical outcomes*			
Rehospitalization	19 (14.4)	141 (16.8)	.563
Recurrent MI	12 (8.1)	5 (0.6)	<.001
Recurrent angina	1 (1.4)	16 (3.2)	.635
MACE^a^	31 (20.1)	53 (5.8)	<.001
*Death rates*			
30‐day mortality	14 (9.1)	4 (0.4)	<.001
1‐year mortality	34 (23.3)	26 (3.1)	<.001
Overall mortality	39 (25.0)	42 (4.6)	<.001

Abbreviations: EF, ejection fraction; LAD, left anterior descending artery; LCX, left circumflex artery; LM, left main; MACE, major adverse cardiovascular events; PCI, percutaneous coronary intervention; RCA, right coronary artery; SVG, Saphenous vein graft.

^a^ MACE was defined as 30‐days mortality, recurrent myocardial infarction, unstable angina, urgent revascularization, stent thrombosis, and cerebrovascular event.

MACE occurred more frequently in the very high‐risk group (20.1% and 5.8%, respectively; *p *< .001), with higher rates of recurrent MIs (8.1% vs. 0.6%, respectively; *p *< .001) and 30‐days mortality (9.1% vs. 0.4%, respectively; *p* < .001). One‐year mortality was also significantly higher in the very‐high risk group (23.3% vs. 3.1%, respectively; *p* < .001).

In a multivariate analysis of the full patient cohort, the very high‐risk category was associated with 1‐year mortality (OR: 7.7, 95% CI: 4.3–13.9; *p* < .001). Age and a history of MI were also significantly associated with a 1‐year mortality rate. However, late PCI (>24 h) was not associated with a higher 1‐year mortality rate (0.96, 95% CI: 0.5–1.8; *p* = .89) (Table S2). In a univariate analysis, a very high‐risk category was significantly associated with 30‐days MACE (OR: 4.06, 95% CI: 2.49–6.53; *p* < .001) (Table S3).

### 3.2 Intragroup analyses

#### 3.2.1 Very high‐risk group

**Figure 1:**
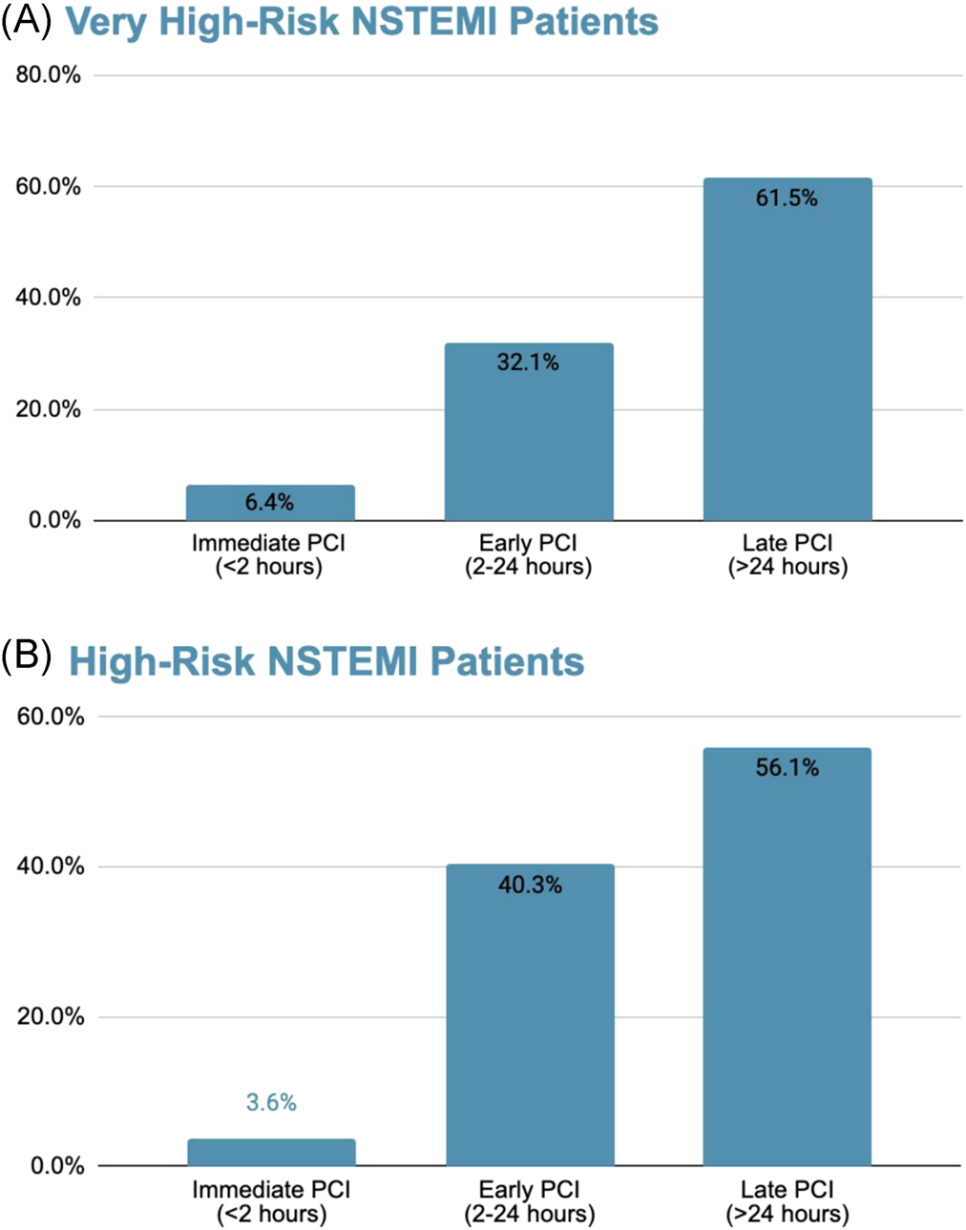
Time to percutaneous coronary intervention in patients with NSTEMI. (A) Very high‐risk patients. (B) High‐risk patients. NSTEMI, non‐ST elevation myocardial infarction; PCI, percutaneous coronary intervention

Out of the 156 patients in the very high‐risk group, only 10 patients (6.4%) underwent immediate coronary angiography, and only 50 (32.1%) patients underwent early coronary angiography. The majority 96 (61.5%) of patients had their PCI performed after more than 24 h (Figure 1A). Overall, the median time to PCI was 35.12 h (IQR: 15.3–71.12) in the very high‐risk NSTEMI group. Baseline characteristics were similar between subgroups (Table 3a). GRACE score ≥ 140 was documented in 6 (75%), 18 (39.1%), and 28 (31.5) of the immediate, early, and late PCI groups, respectively. Patients in the immediate and early coronary angiography subgroups had more ventricular arrhythmias as compared with the late angiography group (4 [11%], 1 [8%], and 0, respectively; *p* < .04). Cardiogenic shock was more common in the immediate and early PCI groups as compared with the late PCI group (4 [40%], 7 [14%], and 3 [3.1%], respectively; *p* < .001).

**Table 3a:** Patients' characteristics in the very high‐risk group

	Immediate PCI	Early PCI	Late PCI	*p*_trend
	10	50	96	
*Baseline characteristics*				
Age, years (median [IQR])	67.00 (61.00, 72.25)	71.00 (66.00, 80.00)	68.00 (63.00, 77.00)	.763
Gender (male)	8 (80.0)	38 (76.0)	60 (62.5)	.072
Dyslipidemia	8 (80.0)	39 (78.0)	72 (75.8)	.697
Hypertension	9 (90.0)	42 (84.0)	83 (87.4)	.844
Current smokers	2 (20.0)	13 (26.0)	28 (29.2)	.502
Diabetes mellitus	8 (80.0)	25 (50.0)	62 (64.6)	.663
Family history of CAD	3 (37.5)	5 (13.5)	18 (23.7)	.889
BMI (kg/m^2^), (median [IQR])	25.11 (21.59, 25.25)	27.76 (25.53, 31.25)	26.97 (24.62, 31.83)	.801
Prior MI	7 (70.0)	24 (48.0)	53 (56.4)	.962
Prior CABG	3 (30.0)	9 (18.0)	13 (13.7)	.187
Prior PCI	5 (50.0)	15 (30.0)	52 (55.9)	.037
Chronic renal failure	2 (20.0)	15 (30.0)	28 (29.5)	.703
Peripheral vascular disease	1 (10.0)	7 (14.0)	15 (15.8)	.61
Cerebrovascular disease	2 (20.0)	9 (18.0)	19 (19.8)	.878
CHF	4 (40.0)	9 (18.0)	24 (25.3)	.929
GRACE score > 140	6 (75.0)	18 (39.1)	28 (31.5)	.03
*Prior medications*				
Aspirin	6 (60.0)	27 (60.0)	60 (69.8)	.271
Clopidogrel	2 (25.0)	6 (15.0)	20 (26.0)	.373
ACE inhibitors	4 (50.0)	12 (32.4)	35 (48.6)	.351
ARB	1 (20.0)	7 (21.2)	19 (30.2)	.338
Beta‐blockers	5 (55.6)	24 (60.0)	54 (64.3)	.524
Statins	7 (77.8)	24 (75.0)	60 (81.1)	.571
Calcium channel blockers	4 (66.7)	14 (42.4)	31 (42.5)	.455
Nitrates	1 (20.0)	1 (3.1)	8 (12.1)	.538
Diuretics	2 (28.6)	7 (20.0)	35 (46.1)	.021
*Vital signs on FMC*				
Admission Killip class				
I	2 (22.2)	24 (48.0)	35 (37.2)	.808
II	3 (33.3)	16 (32.0)	39 (41.5)	.297
III	2 (22.2)	10 (20.0)	18 (19.1)	.822
IV	2 (22.2)	0 (0.0)	2 (2.1)	.062
Heart rate (bpm) (median [IQR])	92.00 (72.00, 107.00)	82.00 (71.00, 103.00)	90.00 (78.00, 106.25)	.207
Systolic blood pressure (mmHg) (median [IQR])	137.00 (117.00, 152.00)	139.00 (120.00, 160.00)	153.00 (128.50, 169.00)	.046
Diastolic blood pressure (mmHg) (median [IQR])	75.00 (65.00, 87.00)	80.00 (71.00, 90.00)	85.00 (72.50, 97.00)	.038
Atrial fibrillation/supraventricular tachycardia	0 (0.0)	3 (6.0)	10 (10.4)	.18
VT/VF	1 (10.0)	4 (8.0)	0 (0.0)	.005
2nd to 3rd degree AV block	0 (0.0)	0 (0.0)	1 (1.0)	.463
*Reperfusion therapy*				
PCI	7 (70.0)	34 (68.0)	62 (64.6)	.623
Coronary angiography	10 (100.0)	50 (100.0)	96 (100.0)	NaN
*In‐hospital complications*				
Mild‐moderate CHF (Killip‐2)	2 (22.2)	17 (34.0)	29 (30.2)	.987
Pulmonary edema (Killip‐3)	1 (11.1)	9 (18.0)	25 (26.0)	.165
Cardiogenic shock (Killip‐4)	4 (40.0)	7 (14.0)	3 (3.1)	<.001
Hemodynamically significant right ventricle infarction	0 (0.0)	1 (2.0)	0 (0.0)	.367
Recurrent MI	0 (0.0)	4 (8.0)	7 (7.3)	.633
Recurrent angina/ischemia	1 (10.0)	1 (2.0)	3 (3.1)	.566
Stent thrombosis	0 (0.0)	0 (0.0)	0 (0.0)	NaN
Free wall rupture	0 (0.0)	0 (0.0)	1 (1.0)	.463
Tamponade	0 (0.0)	0 (0.0)	1 (1.0)	.463
Moderate to severe mitral regurgitation	1 (10.0)	5 (10.0)	7 (7.3)	.581
Pericarditis	0 (0.0)	1 (2.0)	0 (0.0)	.367
Sustained VT (>125 bpm)	1 (10.0)	3 (6.0)	3 (3.1)	.241
Primary VF	0 (0.0)	0 (0.0)	1 (1.0)	.463
Secondary VF	0 (0.0)	1 (2.0)	1 (1.0)	.905
New atrial fibrillation	0 (0.0)	8 (16.0)	7 (7.3)	.574
High degree (2nd to 3rd) AV block	0 (0.0)	1 (2.0)	0 (0.0)	.367
Asystole	0 (0.0)	3 (6.0)	3 (3.1)	.834
Stroke	1 (10.0)	0 (0.0)	0 (0.0)	.011
Acute renal failure	2 (20.0)	10 (20.4)	15 (15.6)	.494
Bleeding	0 (0.0)	2 (4.0)	4 (4.2)	.638
Blood transfusions	0 (0.0)	3 (6.0)	5 (5.2)	.727
*Laboratory tests*				
Peak CK (U/L) value (median [IQR])	670.00 (409.00, 1400.00)	545.00 (305.50, 1328.00)	210.00 (116.00, 475.50)	<.001
Peak troponin I elevated	4 (100.0)	17 (85.0)	30 (85.7)	.625
Peak troponin T elevated	6 (100.0)	32 (94.1)	63 (98.4)	.512
Earliest creatinine (mg/dl) (median [IQR])	1.33 (0.94, 1.80)	1.19 (0.94, 1.51)	1.10 (0.87, 1.46)	.178
*Treatment at discharge*				
Aspirin	8 (100.0)	43 (97.7)	88 (96.7)	.564
P_2_Y_12_	8 (100.0)	40 (93.0)	80 (87.9)	.184
P_2_Y_12_ type				
Prasugrel	2 (20.0)	5 (11.1)	8 (9.0)	.328
Ticagrelor	5 (50.0)	25 (55.6)	33 (37.1)	.076
Clopidogrel	3 (30.0)	15 (33.3)	48 (53.9)	.018
Statins	8 (100.0)	44 (97.8)	90 (98.9)	.855
ACE‐I/ARB	7 (100.0)	38 (92.7)	62 (81.6)	.048
Beta‐blockers	7 (87.5)	38 (88.4)	72 (86.7)	.835
*30‐day clinical outcomes*				
Rehospitalization	2 (25.0)	9 (22.0)	8 (9.6)	.05
Recurrent MI	0 (0.0)	4 (8.3)	8 (8.9)	.462
Recurrent angina	0 (0.0)	0 (0.0)	1 (2.1)	.503
MACE^a^	3 (30.0)	14 (28.0)	14 (14.9)	.053
*Death rates*				
30‐day mortality	2 (20.0)	7 (14.0)	5 (5.3)	.034
1‐year mortality	2 (20.0)	12 (25.0)	20 (22.7)	.959

Abbreviations: ACE, angiotensin‐converting enzyme; ARB, angiotensin receptor blocker; AV, atrioventricular; BMI, Body mass index; CABG, coronary artery bypass grafting; CAD, coronary artery disease; CHF, congestive heart failure; MI, myocardial infarction; PCI, percutaneous coronary intervention; VF, ventricular fibrillation; VT, ventricular tachycardia.

^a^ MACE was defined as 30‐days mortality, recurrent myocardial infarction, unstable angina, urgent revascularization, stent thrombosis, and cerebrovascular event.

#### 3.2.2 Outcomes

MACE occurred in 3 patients (30%) in the immediate group, 14 patients (28%) in the early group, and 14 patients (14.9%) in the late group, (*p* = .053). Mortality rates at 30‐days were 20%, 14%, and 5.3%, respectively (*p* = .034). Mortality rates at 1‐year were 20%, 25%, and 22.7%, respectively (*p* = .959). There was a trend for more recurrent hospitalization in the immediate and early intervention groups as compared with the late intervention group (2 [25%] and 9 [22%] vs. 8 [9.6%], respectively; *p* = .05) (Table 3a).

In a univariate analysis of the very high‐risk patients, late PCI was associated with reduced risk for 30‐days MACE (OR: 0.44; 95% CI: 0.2, 0.98, *p* = .045) (Table S3). This association is not seen when adjusting for the other risk factors (Table S4).

#### 3.2.3 High‐risk group

In the high‐risk group, 33 (3.6%) patients underwent immediate PCI, 372 (40.3%) patients underwent early PCI, and the rest 517 (56.1%) underwent late PCI (Figure 1B). The median time to PCI was 27 h (IQR: 16.6–56.3). Compared with the immediate and early PCI groups, patients in the late PCI subgroup were older (Table 3b). Furthermore, hypertension, dyslipidemia, diabetes, chronic kidney disease, tobacco use, and a family history of ischemic heart disease were more common in the late PCI group. These patients had higher rates of prior MI, PCI, and CABG. Accordingly, these patients were more often chronically treated with antithrombotic, antihypertensive, heart failure, and anti‐ischemic medication. Patients in the late intervention group were less likely to require intervention during coronary angiography (331 [64%] in the late intervention group vs. 25 [75.8%] in the immediate intervention group, and 259 [69.6%] in the early intervention group; *p* = .038). Patients in the late intervention group were more commonly discharged with clopidogrel as compared with patients in the immediate PCI group who were treated more often with prasugrel.

**Table 3b:** Patients' characteristics in the high‐risk group

	Immediate PCI	Early PCI	Late PCI	*p*_trend
	33	372	517	
*Baseline characteristics*				
Age, years (median [IQR])	61.00 (52.00, 72.00)	62.00 (54.00, 71.00)	66.00 (58.00, 74.00)	<.001
Gender (male)	29 (87.9)	306 (82.3)	406 (78.5)	.079
Dyslipidemia	25 (75.8)	272 (73.1)	411 (79.5)	.048
Hypertension	16 (48.5)	233 (62.6)	376 (72.7)	<.001
Current smokers	18 (54.5)	154 (41.4)	183 (35.4)	.011
Diabetes mellitus	8 (24.2)	141 (38.1)	245 (47.4)	<.001
Family history of CAD	13 (44.8)	117 (36.9)	124 (31.1)	.041
BMI (kg/m^2^), (median [IQR])	26.42 (22.93, 28.48)	27.47 (24.54, 30.09)	27.47 (24.75, 30.53)	.214
Prior MI	10 (30.3)	133 (35.8)	236 (45.8)	.001
Prior CABG	3 (9.1)	25 (6.7)	69 (13.3)	.004
Prior PCI	9 (27.3)	112 (30.1)	210 (40.9)	.001
Chronic renal failure	1 (3.0)	22 (5.9)	64 (12.4)	.001
Peripheral vascular disease	1 (3.0)	18 (4.8)	36 (7.0)	.133
Cerebrovascular disease	1 (3.0)	26 (7.0)	47 (9.1)	.126
CHF	2 (6.1)	19 (5.1)	45 (8.7)	.06
GRACE score > 140	6 (19.4)	37 (10.4)	77 (15.4)	.193
*Prior medications*				
Aspirin	12 (38.7)	154 (45.2)	274 (59.3)	<.001
Clopidogrel	3 (10.0)	40 (12.5)	74 (17.1)	.058
ACE inhibitors	6 (21.4)	95 (31.6)	158 (39.5)	.007
ARB	6 (23.1)	56 (21.4)	95 (26.4)	.197
Beta‐blockers	15 (53.6)	114 (37.6)	232 (53.6)	.001
Statins	14 (63.6)	175 (66.8)	292 (78.9)	.001
Calcium channel blockers	4 (14.8)	53 (20.2)	131 (35.6)	<.001
Nitrates	1 (3.8)	10 (4.1)	29 (8.7)	.03
Diuretics	5 (20.0)	27 (10.5)	79 (22.1)	.003
*Vital signs on FMC*				
Admission Killip class				
I	32 (100.0)	356 (100.0)	492 (100.0)	NaN
II–V	0 (0.0)	0 (0.0)	0 (0.0)	NaN
Heart rate (bpm) (median [IQR])	81.50 (72.00, 90.00)	76.00 (66.00, 86.00)	78.00 (68.00, 88.00)	.343
Systolic blood pressure (mmHg) (median [IQR])	142.00 (121.00, 150.00)	147.00 (131.00, 160.00)	147.00 (130.00, 161.00)	.603
Diastolic blood pressure (mmHg) (median [IQR])	83.50 (70.75, 98.00)	83.00 (74.00, 93.00)	81.00 (71.00, 91.00)	.083
Atrial fibrillation/supraventricular tachycardia	1 (3.0)	12 (3.2)	27 (5.2)	.153
VT/VF	0 (0.0)	0 (0.0)	0 (0.0)	NaN
2nd to 3rd degree AV block	0 (0.0)	1 (0.3)	2 (0.4)	.664
*Reperfusion therapy*				
PCI	25 (75.8)	259 (69.6)	331 (64.0)	.038
Coronary angiography	33 (100.0)	372 (100.0)	517 (100.0)	NaN
*In‐hospital complications*				
Mild‐moderate CHF (Killip‐2)	0 (0.0)	0 (0.0)	0 (0.0)	NaN
Pulmonary edema (Killip‐3)	0 (0.0)	0 (0.0)	0 (0.0)	NaN
Cardiogenic shock (Killip‐4)	0 (0.0)	0 (0.0)	0 (0.0)	NaN
Hemodynamically significant right ventricle infarction	0 (0.0)	0 (0.0)	0 (0.0)	NaN
Recurrent MI	0 (0.0)	0 (0.0)	0 (0.0)	NaN
Recurrent angina/ischemia	1 (3.0)	2 (0.5)	10 (1.9)	.283
Stent thrombosis	0 (0.0)	1 (0.3)	1 (0.2)	.949
Free wall rupture	0 (0.0)	0 (0.0)	0 (0.0)	NaN
Tamponade	0 (0.0)	0 (0.0)	0 (0.0)	NaN
Moderate to severe mitral regurgitation	0 (0.0)	0 (0.0)	0 (0.0)	NaN
Pericarditis	0 (0.0)	0 (0.0)	2 (0.4)	.235
Sustained VT ( > 125 bpm)	0 (0.0)	0 (0.0)	0 (0.0)	NaN
Primary VF	0 (0.0)	2 (0.5)	0 (0.0)	.19
Secondary VF	0 (0.0)	0 (0.0)	0 (0.0)	NaN
New atrial fibrillation	0 (0.0)	5 (1.3)	10 (1.9)	.327
High degree (2nd‐3rd) AV block	0 (0.0)	1 (0.3)	2 (0.4)	.664
Asystole	0 (0.0)	0 (0.0)	0 (0.0)	NaN
Stroke	0 (0.0)	2 (0.5)	4 (0.8)	.539
Acute renal failure	1 (3.0)	4 (1.1)	13 (2.5)	.282
Bleeding	0 (0.0)	7 (1.9)	7 (1.4)	.868
Blood transfusions	0 (0.0)	4 (1.1)	7 (1.4)	.512
*Laboratory tests*				
Peak CK (U/L) value (median [IQR])	217.00 (118.00, 558.00)	218.00 (114.75, 459.75)	156.50 (90.50, 337.75)	<.001
Peak troponin I elevated	16 (94.1)	160 (90.9)	200 (88.1)	.268
Peak troponin T elevated	16 (94.1)	213 (94.7)	318 (95.5)	.627
Earliest creatinine (mg/dl) (median [IQR])	0.98 (0.78, 1.09)	0.90 (0.77, 1.03)	0.94 (0.80, 1.12)	.005
*Treatment at discharge*				
Aspirin	33 (100.0)	354 (96.5)	496 (96.5)	.587
P_2_Y_12_	28 (84.8)	341 (93.2)	461 (90.4)	.598
P_2_Y_12_ type				
Prasugrel	9 (30.0)	53 (14.9)	54 (10.8)	.004
Ticagrelor	15 (50.0)	198 (55.6)	251 (50.4)	.249
Clopidogrel	6 (20.0)	105 (29.5)	193 (38.8)	.001
Statins	31 (96.9)	359 (99.4)	489 (98.8)	.856
ACE‐I/ARB	26 (83.9)	278 (83.7)	385 (84.8)	.702
Beta‐blockers	24 (77.4)	266 (80.6)	398 (84.9)	.077
*30‐day clinical outcomes*				
Rehospitalization	5 (17.9)	54 (16.5)	82 (17.0)	.932
Recurrent MI	0 (0.0)	3 (0.9)	2 (0.4)	.578
Recurrent angina	0 (0.0)	5 (2.7)	11 (3.7)	.379
MACE^a^	2 (6.5)	18 (5.0)	33 (6.4)	.472
*Death rates*				
30‐day mortality	0 (0.0)	3 (0.8)	1 (0.2)	.318
1‐year mortality	1 (3.2)	9 (2.6)	16 (3.3)	.637

Abbreviations: ACE, angiotensin‐converting enzyme; ARB, angiotensin receptor blocker; AV, atrioventricular; BMI, Body mass index; CABG, coronary artery bypass grafting; CAD, coronary artery disease; CHF, congestive heart failure; MI, myocardial infarction; PCI, percutaneous coronary intervention; VF, ventricular fibrillation; VT, ventricular tachycardia.

^a^ MACE was defined as 30‐days mortality, recurrent myocardial infarction, unstable angina, urgent revascularization, stent thrombosis, and cerebrovascular event.

#### 3.2.4 Outcome

No difference in MACE rate, mortality, or rehospitalization rate was found between the different intervention groups.

In univariate and multivariate logistic regression models, late PCI was not associated with a higher risk for 30‐days MACE (Tables S3 and S4).

## 4 DISCUSSION

In this nationwide survey, among NSTEMI patients classified as very high‐risk, only 6.4% underwent immediate PCI according to contemporary guidelines, while the majority of them (61.5%) underwent late PCI. Moreover, among patients with high‐risk NSTEMI, only 43.9% underwent immediate or early PCI, while 56.1% of patients underwent late PCI. Our findings unequivocally demonstrate a large gap between the guidelines' recommendation and the daily practice treatment in a nationwide survey.

There are several explanations for these findings. First is the lack of consistent evidence for improved outcomes in immediate and early intervention. Very high‐risk patients have generally been excluded from randomized controlled trials; hence, the recommendation for immediate PCI (class IC) is largely based on the fact that these patients have a poor prognosis with conservative treatment.^13^ Among the high‐risk group, there are a number of randomized controlled trials addressing the issue; however, the data are inconclusive. One of the reasons is the heterogeneity of these studies. Importantly, the actual time to intervention in the early intervention group varies widely between the studies. For example, the studies on which the guidelines are based did not necessarily examine only 24 h as a definition for early catheterization but included studies in which the definition for early intervention was longer.^5^ Another explanation might be the availability of interventional teams worldwide, which are not on‐site 24/7; hence, the ability and desire to perform early coronary angiography to all NSTEMI patients admitted outside the regular working hours are not optimal.^16^ Another important possible explanation is the paradigm shift embedded in the new guidelines. In the past, there was an opposite approach of waiting several days for “cooling” of the infarct in NSTEMI patients.^5^, ^17^ The idea stemmed from the fear of embolization of nonocclusive thrombus overlying the ruptured plaque, which may cause a periprocedural MI or consequent slow/no‐reflow phenomenon if immediate PCI is undertaken in such lesions.^5^ Another option, and perhaps the most important one, is the tendency to postpone PCI in complicated and unstable patients such as old patients with several comorbidities (i.e., diabetes mellitus and renal failure) to allow stabilization and bring them in an optimally hemodynamic and respiratory condition for PCI. In these patients, there is, sometimes, an operator preference to postpone PCI for the morning hours when additional staff is present, in case a complication occurs during the procedure.^18^ Importantly, the fact that the study is a nationwide study also suggests that these findings do not reflect an independent practice of a single‐center but rather reflect a broader conceptual attitude toward the NSTEMI guidelines.

There is ample evidence of the prognostic importance of complying with medical guidelines.^19^, ^20^, ^21^ Moreover, studies have shown that in NSTEMI patients, adherence to guidelines reduces mortality in the first 3 years after infarction.^22^ The fact that compliance with the guidelines is so low, even in a small country where every hospital has PCI capabilities, emphasizes the difficulty of meeting these standards, at least when it comes to PCI timing.

Our study has several limitations: First, the study is an observational study and, as such, is subjected to confounding factors. For example, there is naturally a selection bias in choosing whom to proceed with early PCI. This can be seen in the data—the high‐risk NSTEMI patients with the delayed PCI were older and suffered more from comorbidities, which probably influenced the decision of the operator to delay the procedure. Second, there was no difference in the mortality rate or MACE between the various intervention groups. However, the study was not powered to demonstrate such differences. The main strengths of our study are the fact that it is based on real‐world data and represents a nonselected consecutive NSTEMI patient population. Furthermore, it is based on a multicenter nationwide; therefore, the results do not reflect a single‐center approach and are more generalizable.

## 5 CONCLUSION

In conclusion, our data suggest that despite the recent guidelines recommendations for immediate and early PCI in very high‐risk and high‐risk NSTEMI patients, respectively, most patients do not undergo immediate or early PCI. Further studies are needed to better understand the reasons for guidelines' nonadherence in those high‐risk patients.

## Supporting information

Supporting information.

## Data Availability

Data available on request from the authors.
