# Supplementary material for: Immediate and early percutaneous coronary intervention in very high‐risk and high‐risk non‐ST segment elevation myocardial infarction patients
Source: Clin Cardiol. 2022 Mar 9;45(4):359–69. doi: 10.1002/clc.23781 (PMC9019882; doi:10.1002/clc.23781)
Supplement: Supplementary file 1 — Supporting information. [file CLC-45-359-s001.docx]

Supplementary

**Supplementary Table 1. Risk factors prevalence in each risk level category***

|  | n (%) |
| --- | --- |
| **Very high-risk (%)** | **156 (14.1)** |
| Hemodynamic instability and cardiogenic shock (Killip class 4) | 15 (1.4) |
| Ongoing or recurrent pain refractory to medical treatment | 11 (1.0) |
| Life-threatening arrhythmia or cardiac arrest | 25 (2.3) |
| Mechanical complication (free wall rupture, tamponade, ventricular septal defect, moderate to severe mitral regurgitation) | 14 (1.3) |
| Acute heart failure (Killip class 2 or 3) | 124 (11.2) |
| **High-risk (%)** | **1076 (98.7)** |
| Rise or fall in troponin compatible with myocardial infarction | 1070 (96.4) |
| Dynamic ST-segment or T-wave changes | 18 (1.6) |
| GRACE score higher than 140 | 172 (15.5) |

***** Patients who had risk factors for more than one category were classified to the higher risk category.

**Supplemntary Table 2. Multivariate logistic regression for the prediction
of one-year mortality in the all cohort**

| **Variable** | **Odds ratio (95% CI)** | **p-value** |
| --- | --- | --- |
| Age | 1.07 (1.04, 1.10) | **<0.001** |
| Male | 0.93 (0.49, 1.84) | 0.84 |
| Diabetes | 1.01 (0.55, 1.85) | 0.99 |
| Prior MI | 2.48 (0.99, 6.06) | **0.05** |
| Prior CABG | 0.98 (0.41, 2.15) | 0.96 |
| Prior PCI | 0.76 (0.33, 1.85) | 0.54 |
| Very high-risk category | 7.67 (4.27, 13.91) | **<0.001** |
| Late PCI (>24 hours) | 0.96 (0.52, 1.77) | 0.89 |

CI, confidence interval; MI, myocardial infarction; CABG, coronary
Artery bypass grafting; PCI, percutaneous coronary intervention

**Supplementary Table 3. Univariate logistic regression model for the prediction of 30-days MACE**

| **Variable** | **All Cohort** | | **High-risk patients** | | **Very high-risk patients** | |
| --- | --- | --- | --- | --- | --- | --- |
|  | **Odds ratio (95% CI)** | **p-value** | **Odds ratio (95% CI)** | **p-value** | **Odds ratio (95% CI)** | **p-value** |
| Age | 1.02 (1,1.04) | **0.047** | 1.02 (1,1.04) | 0.099 | 0.99 (0.95,1.03) | 0.527 |
| Gender (male) | 0.62 (0.38,1.02) | 0.054 | 0.59 (0.33,1.14) | 0.1 | 1.01 (0.44,2.43) | 0.978 |
| Diabetes | 1.21 (0.78,1.9) | 0.395 | 0.87 (0.48,1.52) | 0.617 | 1.44 (0.64,3.43) | 0.393 |
| Family history of CAD | 0.88 (0.5,1.5) | 0.653 | 1 (0.5,1.92) | 0.99 | 1.11 (0.37,3.01) | 0.844 |
| Prior MI | 1.1 (0.7,1.73) | 0.665 | 1.02 (0.57,1.78) | 0.943 | 0.86 (0.39,1.91) | 0.708 |
| Prior CABG | 1.34 (0.67,2.46) | 0.375 | 0.88 (0.3,2.08) | 0.797 | 1.69 (0.6,4.36) | 0.296 |
| Prior PCI | 1.02 (0.64,1.61) | 0.918 | 1.08 (0.6,1.9) | 0.785 | 0.65 (0.29,1.45) | 0.3 |
| Late PCI (>24 hours) | 0.95 (0.61,1.49) | 0.814 | 1.29 (0.73,2.32) | 0.385 | 0.44 (0.2,0.98) | **0.045** |
| Very high-risk (vs. high risk) | 4.06 (2.49,6.53) | **<0.001** |  |  |  |  |

CI, confidence interval; CAD, coronary artery disease; MI, myocardial infarction; CABG, coronary Artery bypass grafting; PCI, percutaneous coronary intervention

**Supplementary Table 4. Multivariate logistic regression model for the prediction**

**of 30-days MACE**

| **Variable** | **High-risk patients** | | **Very high-risk patients** | |
| --- | --- | --- | --- | --- |
|  | **Odds ratio (95% CI)** | **p-value** | **Odds ratio (95% CI)** | **p-value** |
| Age | 1.02 (0.99, 1.04) | 0.18 | 0.98 (0.94, 1.02) | 0.3 |
| Gender (male) | 0.66 (0.35, 1.31) | 0.22 | 0.85 (0.34, 2.19) | 0.73 |
| Diabetes | 0.77 (0.42, 1.37) | 0.38 | 1.73 (0.71, 4.47) | 0.24 |
| Prior MI | 0.96 (0.36, 2.39) | 0.93 | 1.18 (0.30, 4.42) | 0.82 |
| Prior CABG | 0.86 (0.28, 2.22) | 0.78 | 1.61 (0.50, 4.91) | 0.41 |
| Prior PCI | 1.18 (0.47, 3.06) | 0.74 | 0.50 (0.13, 2.01) | 0.33 |
| Late PCI (>24 hours) | 1.22 (0.69, 2.22) | 0.51 | 0.48 (0.20, 1.17) | 0.11 |

CI, confidence interval; CAD, coronary artery disease; MI, myocardial infarction;
CABG, coronary Artery bypass grafting; PCI, percutaneous coronary intervention
